# Supplementary material for: WordCommentsAnalyzer: A windows software tool for qualitative research
Source: F1000Res. 2018 Sep 4;7:536. Originally published 2018 May 3. [Version 2] doi: 10.12688/f1000research.14819.2 (PMC6137411; doi:10.12688/f1000research.14819.2)
Supplement: Supplementary file 3 [file f1000research-7-17614-s0002.tgz › b2a79496-4ada-47c4-9682-a99ce867836d.zip!/Eight Iranian Health Journals/The Journal of Toloo-e-Behdasht_31.docx]

© The Journal of Toloo-e-Behdasht. This work is licensed under CC BY 4.0 (https://creativecommons.org/licenses/by/4.0/).

Volume & Issue: دوره 12 - شماره 1

Title: بررسی ترکیب شیمیایی و خواص ضد باکتریایی اسانس رزماری بر روی اشرشیاکلی و تعیین سینتیک آن

http://tbj.ssu.ac.ir/article-1-453-fa.html

مقدمه: به علت مقاومت میکروارگانیسمها در برابر آنتی بیوتیک ها، استفاده از گیاهان دارویی جهت دستیابی به ترکیبات جدید و غلبه بر آنها، امری مهم و ضروری می باشد. هدف از انجام این مطالعه، بررسی ترکیبات موثر و خواص ضد باکتریایی اسانس رزماری بر روی اشرشیاکلی، به عنوان یکی از شایعترین باکتریهای بیماریزا و تعیین سینتیک اثر آن می باشد.   روش بررسی: پس از جداسازی گیاه رزماری از دیگر گیاهان اقلیم شهر کرمان و خشک نمودن آن، استخراج اسانس به روش تقطیر با آب انجام گردید. اسانس بدست آمده، بوسیله گازکروماتوگرافی جرمی آنالیز و ترکیبات موجود در آن شناسایی شد.جهت تعیین خواص ضد باکتریایی، از روشهای استاندارد انتشار دیسک، تعیین حداقل غلظت ممانعت کنندگی و تعیین حداقل غلظت کشندگی استفاده شد. ضمنا سینتیک اثر اسانس بر روی باکتری نیز تعیین گردید.   یافته ها: در آنالیز اسانس رزماری، 20 ترکیب شناسایی شد که 09/82 درصد این ترکیبات شامل 1,8 Cineole ، Borneo ، -pinen α ، Camphor ، Linalool ، Camphene ، limonene و Verbenone می باشد. قطر هاله عدم رشد بدست آمده در غلظت µg/Disc 32 اسانس، از قطر هاله عدم رشد آنتی بیوتیک های جنتامایسین، پنی سیلین، استرپتومایسین و اریترومایسین بیشتر می باشد. همچنین حداقل غلظت ممانعت کنندگی از رشد( MIC ) و حداقل غلظت کشندگی ( MBC ) در این مطالعه به ترتیب برابر 3000 و 3200 حاصل شد. زمان مورد نیاز برای نابودی کامل اشرشیاکلی نیز 25 دقیقه بدست آمد.   نتیجه گیری: بررسی های انجام گرفته در این مطالعه نشان داد اسانس گیاه رزماری جمع آوری شده از شهر کرمان دارای خاصیت ضدباکتریایی بسیار خوبی بر علیه اشرشیاکلی بوده، بطوریکه نسبت به برخی آنتی بیوتیکهای دارویی رایج، این خاصیت را در غلظت کمتری، نشان می دهد.

Title: اثر مکمل کوآنزیم Q10 بر فشارخون بیماران مبتلا به پرفشاری خون: یک مطالعه کارآزمایی بالینی تصادفی دو سو بی خبر

http://tbj.ssu.ac.ir/article-1-454-fa.html

مقدمه: پرفشاری خون یکی از مهمترین چالشهای سلامت بشر در حال حاضر و از مهمترین عوامل خطرساز بیماریهای قلبی-عروقی و کلیوی است. درمان و کنترل این مشکل به کمک روش های نوین و کم عارضه از اهمیت ویژه ای برخوردار است. از این رو مطالعه حاضر با هدف تعیین اثر مکمل کوآنزیم Q10 بر فشارخون بیماران مبتلا به پرفشاری خون انجام گردید.   روش بررسی: این کارآزمایی بالینی تصادفی شده با طول دوره مکمل یاری سه ماهه با مشارکت 60 نفر از بیماران مبتلا به پرفشاری خون انجام شد. بیماران به طور تصادفی به دو گروه آزمون و دارو نما تقسیم، که گروه آزمون روزانه یک کپسول حاوی 100 میلی گرم کوآنزیم Q10 خوراکی و گروه دارونما روزانه یک کپسول مشابه فاقد مکمل دریافت کردند. قبل از شروع مداخله و پس از دوازده هفته فشارخون سیستول و دیاستول اندازه گیری شد.   یافته ها: میانه فشارخون سیستول قبل و بعد از مداخله در گروه آزمون به ترتیب از 150 به 130 میلیمتر جیوه رسید (002/0 P = ). این ارقام در گروه دارونما به ترتیب 150 و 147(52/0 P = ) بدست آمده است. میانه فشار خون سیستول قبل از مداخله بین دو گروه تفاوت معنی داری نشان نداده ولی در پایان مداخله این دو گروه تفاوت معنی داری داشته اند (01/0 P = ). میانه فشارخون دیاستول قبل و بعد از مداخله بین دو گروه تفاوت معنی داری نشان نداده و در هر گروه نیز قبل و بعد تفاوت معنی داری مشاهده نشده است.   نتیجه گیری: این مطالعه نشان داد که مصرف روزانه 100 میلی گرم کوآنزیم Q10 طی 12 هفته همراه با درمان دارویی معمول در بیماران مبتلا به پرفشاری خون خفیف به کاهش معنی دار فشارخون سیستولی منجرمی شود. بنا بر این استفاده از آن به عنوان مکمل تکمیلی توصیه می شود.

Title: مطالعه استرس شغلی رانندگان شرکت واحد اتوبوسرانی شهر همدان در سال 1390

http://tbj.ssu.ac.ir/article-1-1810-fa.html

مقدمه: امروزه استرس تبدیل به یک پدیده جهانی رو به رشدی شده است که تمامی گروههای شغلی و محیط‌های کاری را در تمام کشورها تحت تأثیر قرار می‌دهد. بر طبق مطالعات، رانندگی اتوبوس یکی از موقعیت‌های پر‌استرس شغلی می‌باشد. استرس می‌تواند از طریق تداخل با عملکرد ایمن راننده، منجر به بروز حادثه شود. این مطالعه با هدف بررسی وضعیت استرس شغلی رانندگان اتوبوس‌های شرکت واحد شهر همدان انجام شد. روش بررسی: این مطالعه مقطعی در بهار سال 1390 انجام شد. جامعه آماری، کلیه رانندگان شرکت واحد اتوبوس‌رانی شهر همدان بودند. تعداد 54 نفر با استفاده از روش نمونه‌گیری تصادفی ساده انتخاب شدند. به منظور جمع‌آوری اطلاعات از پرسشنامه استرس شغلی فیلیپ ال رایس مورد استفاده قرار گرفت. آزمون‌های آماری مورد استفاده در تحلیل داده‌ها، شامل t-test و ANOVA بود. یافته‌ها: نتایج نشان داد 76% از رانندگان سطح استرس بالا، 16% سطح استرس متوسط و 8% سطح استرس پائین دارند و بیشترین همبستگی نمره استرس شغلی به ترتیب با روابط بین فردی و استرس فیزیکی به دست آمد. در این مطالعه رابطه استرس شغلی با خصوصیات دموگرافیک رانندگان از لحاظ آماری معنی‌دار نبود (05/0<P). نتیجه‌گیری: سطح بالای استرس رانندگان، ضرورت اجرای یک برنامه جامع مدیریت استرس را در سطح سازمان نشان می‌دهد. رسیدگی به وضعیت ارتقاء و امنیت شغلی و برقراری امکان ارتباط مؤثر رانندگان با سرپرستان و فراهم کردن امکان مشارکت آنها در تصمیم‌گیری‌ها بایستی در اولویت اول با هدف بهبود وضعیت استرس شغلی رانندگان قرار‌گیرد.

Title: بررسی کارآیی روش وتلند مصنوعی زیرسطحی در تصفیه فاضلاب شهر یزد در سال 1390

http://tbj.ssu.ac.ir/article-1-457-fa.html

مقدمه: استفاده از سیستم های تصفیه فاضلاب طبیعی با تکنولوژی پایین مانند وتلند های مصنوعی علاوه بر کاهش هزینه های اقتصادی به اصلاح محیط زیست نیز کمک می کنند. هدف از این مطالعه تعیین کارآیی روش وتلند مصنوعی زیرسطحی در تصفیه فاضلاب شهر یزد بوده است.   روش بررسی: مطالعه حاضر یک پژوهش مداخله ای آزمایشگاهی است که در آن کارایی روش وتلند زیرسطحی در حذف پارامترهای , PO4-P ,NH3-N ,NO3-N ,TSS , BOD5 , COD کلیفرم کل و کلیفرم گوارشی مورد بررسی قرار گرفت. بدین منظور 2 راکتور بصورت پایلوت به روش وتلند مصنوعی با جریان زیر سطحی با زمان ماند چهار روز ساخته شد. طی 2 ماه نمونه ها از ورودی و خروجی راکتورها برداشته شد و مطابق با روش های استاندارد و با استفاده از نرم افزار آماری SPSS ver 11.5 و Excel 2003 تجزیه و تحلیل شد.   یافته ها: طبق نتایج بدست آمده راندمان حذف پارامترهای , PO4-P ,NH3-N ,NO3-N ,TSS , BOD5 , COD کلیفرم کل و کلیفرم گوارشی در وتلند گیاه نخل مرداب به ترتیب برابر با 74، 73، 84، 40، 36، 33،70 و 38 درصد ودر وتلند شاهد به ترتیب برابر با 44، 34، 77، 15، 3/0، 1، 17 و 26 درصد بوده است.   نتیجه گیری: با توجه به یافته های مطالعه، وتلند مصنوعی با جریان زیر سطحی در حذف مواد آلی و جامدات دارای راندمان بالایی بوده و قادر به دستیابی به استانداردهای زیست محیطی ایران برای مصارف کشاورزی و آبیاری می باشد. همچنین این گیاه راندمان مناسبی در حذف مواد مغذی و کلیفرم کل و کلیفرم گوارشی بوده است. البته لازم است بررسی های بیشتری بر روی کارآیی این روش در حذف توترینت ها، فلزات سنگین، تخم انگل و ویروس های روده ای انجام گیرد.

Title: استرس اقتصادی، شادکامی وکیفیت زندگی در بیماران 20 تا 35 ساله سرطانی کاشمر

http://tbj.ssu.ac.ir/article-1-458-fa.html

مقدمه: سرطان به عنوان سومین عامل مرگ پس از بیماری های قلبی و تصادفات در ایران شناخته شده است، به همین علت همواره جزء اولویت های تحقیقاتی قراردارد. پژوهش حاضر با هدف بررسی رابطه بین استرس اقتصادی با شادکامی و کیفیت زندگی در بیماران 20 تا 35 ساله سرطانی شهرستان کاشمر اجرا گردید.   روش بررسی: نمونه تحقیق را 60 نفر از بیماران سرطانی ساکن کاشمر تشکیل داد. از این تعداد 59 درصد را مردان و 41 درصد را زنان تشکیل می دادند که به شیوه نمونه گیری دردسترس از جامعه مورد نظر انتخاب شدند. پرسشنامه های مورد نظر اجرا، نتایج جمع آوری و با استفاده از تحلیل رگرسیون چند متغیری مورد تجزیه وتحلیل قرار گرفت.   یافته ها: یافته های پژوهش در باره ارتباط استرس اقتصادی و شادکامی در بیماران سرطانی نشان داد که استرس اقتصادی (خرده مقیاس پول پرسشنامه فقر اقتصادی) و استرس اقتصادی فعلی با شادکامی ارتباط معکوس دارند، طوری که افزایش یک واحد در استرس اقتصادی (خرده مقیاس پول پرسشنامه فقر اقتصادی) و استرس اقتصادی فعلی به ترتیب به اندازه 41/0 و 34/0 شادکامی را کاهش می دهد.   یافته های پژوهش در باره ارتباط استرس اقتصادی و کیفیت زندگی در بیماران سرطانی نشان داد که استرس اقتصادی (خرده مقیاس پول پرسشنامه فقر اقتصادی) و استرس اقتصادی فعلی با کیفیت زندگی ارتباط معکوس دارند، طوری که افزایش یک واحد در استرس اقتصادی (خرده مقیاس پول پرسشنامه فقر اقتصادی) و استرس اقتصادی فعلی به ترتیب به اندازه 42/0 و 27/0 کیفیت زندگی را کاهش می دهد.   نتیجه گیری: آنچه از جمع بندی نتایج بدست آمده از پژوهش های انجام شده و این پژوهش حاصل شد این است که داده های بدست آمده از بیماران سرطانی نشان داده که تشخیص، درمان و هزینه های سرطان درماندگی های روانشناختی قابل توجهی بوجود می آورد و این بر کیفیت زندگی و شادکامی این بیماران تاثیر منفی دارد.

Title: بررسی میزان تاثیر بکارگیری الگوی اعتقاد بهداشتی در اتخاذ رفتارهای پیشگیری و کنترل کننده‌ی بهداشت دوران بلوغ در دانش آموزان دختر مقطع متوسطه شهرستان اردکان

http://tbj.ssu.ac.ir/article-1-459-fa.html

مقدمه: بلوغ فرایند رشدی مهم و مرحله ای از زندگی می باشد. دختران نیازهای مراقبتی بهداشتی متعددی دارند و باید در مورد موضوعات خاص خودشان مورد توجه قرار گیرند. بهداشت بلوغ منجر به حفظ و ارتقاء سلامت روانی، جسمی و عاطفی فرد می شود. این مطالعه با هدف بررسی بکارگیری الگوی اعتقاد بهداشتی در اتخاذ رفتارهای پیشگیری و کنترل کننده ی بهداشت بلوغ در دانش آموزان دختر مقطع متوسطه شهرستان اردکان انجام شد.   روش بررسی: این مطالعه بصورت کارآزمایی بالینی تصادفی از نوع مداخله ای و غیر مداخله ای انجام پذیرفت. جامعه مورد بررسی، دانش آموزان مقطع اول متوسطه شهرستان اردکان و نمونه گیری به صورت تصادفی خوشه ای بود. تعداد نمونه با توجه به نتایج مطالعات مشابه 120 نفر تخمین زده شد. داده ها به وسیله پرسشنامه پیش ساخته جمع آوری گردید برای اندازه گیری اعتبار پرسشنامه از روش اعتبار محتوی و پایایی از طریق آزمون مجدد استفاده شد و داده ها توسط نرم افزار SPSS و با استفاده از شاخص های مرکزی و پراکندگی، تی زوجی، تی مستقل، کای اسکوئر، ضریب همبستگی اسپیرمن و آنالیز واریانس تجزیه و تحلیل شد.   یافته ها: بعد از مداخله، میانگین نمره آگاهی، سازه های الگوی اعتقاد بهداشتی و رفتار در گروه مداخله و غیر مداخله تفاوت معنی دار آماری داشت، ولی در میانگین نمره راهنمای عمل، اختلاف معنی دار مشاهده نشد. بین میانگین نمره آگاهی، سازه های الگوی اعتقاد بهداشتی و رفتار قبل و بعد از مداخله آموزشی در گروه شاهد اختلاف معنی دار آماری وجود داشت(00/0 p= )، اما بین میانگین نمره حساسیت درک شده، راهنمایی برای عمل، اختلاف معنی دار مشاهده نشد. همچنین بین میانگین نمره آگاهی، سازه های الگوی اعتقاد بهداشتی و رفتار قبل و بعد از مداخله آموزشی در گروه مورد اختلاف معنی دار آماری مشاهده شد(00/0 p= ).   بحث و نتیجه گیری : یافته های این بررسی تاثیر و کارایی مدل اعتقاد بهداشتی را در اتخاذ رفتارهای پیشگیری و کنترل کننده در دختران سنین بلوغ، درباره بهداشت این دوران راتایید می کند.

Title: بررسی کارایی فرایند شبه فنتون در حذف 4-کلروفنل از محیطهای آبی

http://tbj.ssu.ac.ir/article-1-460-fa.html

مقدمه: 4-کلروفنل یکی از انواع کلروفنل ­ هایی است که در پساب صنایعی اعم از پالایشگاه­ها، صنایع پتروشیمی، صنایع تولید حشره­کش و.....یافت شده و سبب آسیب به محیط زیست می شود. هدف از انجام این تحقیق بررسی کارایی فرایند Fe°/H2O2 در تجزیه 4-کلروفنل از محلولهای آبی و بررسی ترکیبات واسطه تولیدی حاصل از واکنش بوده است.   روش بررسی: این مطالعه یک پژوهش کاربردی است که به صورت تجربی و در مقیاس آزمایشگاهی انجام شد. در طول آزمایش مقادیر بهینه پارامترهای مختلف از قبیل pH ، پراکسید هیدروژن، پودرآهن، زمان بهینه وسرعت اختلاط در حذف4- کلروفنل بررسی شد. به منظور بررسی ترکیبات واسطه تولیدی، آزمایشات کلر باقیمانده، UV215 و COD نیز انجام گرفت.   یافته ها: در این تحقیق pH بهینه 4 تعیین شد و همچنین نسبت های مولی بهینه پراکسید هیدروژن بر 4-کلروفنل برابر با 8/18، پراکسید هیدروژن بر پودرآهن برابر با 4/0، زمان بهینه 5 دقیقه و سرعت اختلاط rpm 180 بدست آمد. در نهایت بمنظور بررسی میزان معدنی شدن 4-کلروفنل از شاخص COD استفاده شد که نشان داد، ترکیب4-کلروفنل به طور کامل معدنی نشده است. در عین حال افزایش میزان غلظت یون کلرور وسنجش UV215 پس از زمان بهینه 5 دقیقه نشان داد خاصیت کلرینه و حلقه بنزنی ترکیب اولیه کاهش یافته، که این امر نشان دهنده بهبود تجزیه پذیری 4-کلروفنل بوده است.   نتیجه گیری : با توجه به کلیه نتایج بدست آمده و در نظر گرفتن مقاومت بالای ترکیب 4-کلروفنل در برابر تجزیه توسط فرایندهای بیولوژیکی، می توان از فرایند Fe°/H2O2 برای تبدیل نمودن این ترکیب به سایر ترکیباتی آلی که قابلیت تجزیه پذیری بیولوژیکی بالاتر و سمیت کمتری داشته باشند استفاده نمود.

Title: بررسی رفتارهای پیشگیری کننده از عفونت ادراری بر اساس الگوی اعتقاد بهداشتی (HBM) در مادران دارای دختر کمتر از 6 سال

http://tbj.ssu.ac.ir/article-1-461-fa.html

مقدمه :عفونت ادراری دومین عفونت شایع در کودکان است . در سال اول زندگی شیوع آن در پسران بیشتر بوده و بعد از آن در تمام دوران کودکی در دختران بیشتر است. از عوارض حاصل از عفونت ادراری، پرفشاری خون، کاهش عملکرد کلیه، پروتئین اوری و در نهایت بیماری های کلیوی می باشد .   روش بررسی :مطالعه حاضر به صورت توصیفی تحلیلی بر روی 138 نفر ازمادران دارای دختر کمتر از 6 سال در سال 91-1390 انجام گرفت.اطلاعات توسط پرسشنامه وبصورت تصادفی جمع آوری گردید وبا استفاده از نرم افزار SPSS مورد تجزیه و تحلیل قرار گرفت.   یافته ها: نتایج نشان داد که نمره آگاهی تنها در9/2% موارد مطلوب بود. حساسیت درک شده در 7/58% موارد ، شدت درک شده در 7/66% موارد، موانع درک شده در 2/65% موارد ،مطلوب گزارش گردید . بین حساسیت ، شدت ،رفتار و خودکارآمدی همبستگی مستقیم وجود داشت .همچنین بین شدت،تهدید ، راهنمای عمل و خودکارآمدی،بعلاوه بین شدت و خودکارآمدی و بین راهنمای عمل،رفتار و خودکارآمدی و بین رفتارو خودکارآمدی همبستگی مستقیم وجود داشت و بین موانع، حساسیت،منافع،شدت،راهنمای عمل و خودکارآمدی همبستگی معکوس معنی داری وجود داشت و بین شدت و سن مادر، بین تهدید و شدت، بین رفتار وخودکارآمدی و تعداد فرزندان، بین رفتار و وضعیت اشتغال مادر ،بین راهنمای عمل و خودکارآمدی و میزان درآمد خانواده وبین راهنمای عمل، رفتارو خودکارآمدی با سابقه ابتلای کودک به عفونت ادراری ، رابطه معنی داری مشاهده شد.   نتیجه گیری: با توجه به میزان نمرا ت حاصل از سازه ها،پیشنهاد می شود تا برنامه های آموزشی مبتنی برالگوی اعتقاد بهداشتی به منظور بهبود رفتارهای پیشگیری کننده از عفونت ادراری صورت گیرد.

Title: تاثیر آموزش مولفه های هوش هیجانی بر افزایش شادکامی دانشجویان دختر خوابگاهی

http://tbj.ssu.ac.ir/article-1-462-fa.html

مقدمه : هوش هیجانی از پنج مولفه درون فردی، بین فردی، انطباق پذیری، مدیریت استرس و خلق کلی تشکیل شده است . سطح پایین هوش هیجانی را می توان از طریق آموزش افزایش داد. محققان معتقدند که شادکامی، شامل حالت خوشحالی و یا سرور (هیجانات مثبت)، راضی بودن از زندگی و فقدان افسردگی و اضطراب (عواطف منفی) است.هدف از این پژوهش بررسی تأثیر آموزش هوش هیجانی بر افزایش شادکامی دختران خوابگاهی است.   روش بررسی : این پژوهش با روش کارآزمایی بالینی تصادفی شده انجام شد . که دارای دو گروه است و هر دو گروه دو بار مورد ارزیابی قرار گرفته اند. نخستین ارزیابی با اجرای یک پیش آزمون و دومین ارزیابی با اجرای یک پس آزمون صورت گرفت که گروه آزمایش مورد مداخله 10جلسه آموزش مولفه های هوش هیجانی به مدت 2 ساعت قرار گرفتند و باگروه کنترل مقایسه شدند . 443 نفر از دانشجویان دختر خوابگاهی دانشگاه آزاد اسلامی واحد یزد در سال 1390 با روش نمونه گیری مناسب انتخاب شدند. در ادامه از بین دانشجویانی که نمره شادکامی آنها از میانه پایین تر بود 60 نفر به طور تصافی انتخاب شدند (30 نفر گروه کنترل و 30 نفر گروه آزمایش).   یافته ها : تحلیل داده ها نشان می دهد که میانگین تفاضل نمرات شادکامی پیش آزمون و پس آزمون گروه آزمایش با میانگین تفاضل نمرات شادکامی پیش آزمون و پس آزمون گروه کنترل تفاوت معنی دار دارد ( 001 p   نتیجه گیری : آموزش مولفه های هـوش هیجانی باعث می شود تا فرد، بـهـتر بتواند با مسایلی که ویژه محیـط های خوابگاهی است، کنار آید و از شادکامی بالا تری برخوردار شود.

Title: بررسی کاربرد الگوی اعتقاد بهداشتی درتبیین انجام غربالگری سرطان دهانه رحم در زنان 20 تا 65 ساله شهرستان لردگان چهار محال بختیاری

http://tbj.ssu.ac.ir/article-1-463-fa.html

مقدمه : سرطان دهانه رحم دومین سرطان شایع بعد از سرطان پستان در دنیا می‌باشد. ترغیب زنان جامعه به شرکت در برنامه‌های غربالگری سرطان دهانه رحم موجب تشخیص، درمان به موقع و کاهش عوارض و مرگ ناشی از این بیماری می‌گردد. الگوی اعتقاد بهداشتی، به عنوان یک الگوی اکتشافی جهت ارزیابی اینکه چرا افراد از خدمات پیشگیرانه استفاده نمی کنند در تحقیقات متعددی بکار گرفته شده است. هدف از این مطالعه تعیین کاربرد الگوی اعتقاد بهداشتی در انجام غربالگری سرطان دهانه رحم در زنان 20 تا 65 ساله شهرستان لردگان استان چهار محال بختیاری بود.   روش بررسی : این مطالعه توصیفی تحلیلی بصورت مقطعی بر روی زنان 20 تا 65 ساله شهرستان لردگان انجام شد. تعداد نمونه 300 زن ازدواج کرده بودند که به صورت تصادفی خوشه‌ای انتخاب شدند. ابزار گردآوری داده‌ها پرسشنامه‌ای بود که از زیر مقیاس‌های اطلاعات شخصی، آگاهی، حساسیت درک شده، شدت درک شده، منافع درک شده، موانع درک شده و راهنما برای عمل تشکیل شده بود. برای اندازه گیری اعتبار پرسشنامه از روش اعتبار محتوا و پایایی آن از آزمون- بازآزمون استفاده شد. تجزیه تحلیل داده‌ها توسط نرم افزار SPSS نسخه 5/11 و با استفاده از شاخص‌های مرکزی و پراکندگی، ضریب همبستگی پیرسون، رگرسیون لوجستیک، رگرسیون خطی و آنالیز واریانس صورت گرفت.   یافته‌ها :میانگین نمره آگاهی 75/2±12/7، حساسیت درک شده 09/1±45/3، شدت درک شده 45/1±92/5، منافع درک شده 22/1±33/2، موانع درک شده 60/3±14/14 و راهنما برای عمل 05/1±04/1 بود. تنها 37 درصد از افراد مورد مطالعه گزارش نمودند که تابحال تست پاپ اسمیر را انجام داده‌اند. میانگین تعداد دفعات انجام تست پاپ اسمیر در این افراد 01/1±58/0 بدست آمد. بر اساس نتایج آنالیز رگرسیون لوجستیک فقط آگاهی(017/0 p= ) و موانع درک شده (025/0 p= ) به طور معنی داری انجام تست پاپ اسمیر را پیش بینی نمودند، بعلاوه منافع درک شده به طور معنی داری تعداد دفعات انجام تست پاپ اسمیر را پیش بینی نمود (04/0 p= ).   نتیجه گیری : نظر به اینکه بخش محدودی از سازه‌های الگوی اعتقاد بهداشتی دفعات و یا انجام تست پاپ اسمیر را پیش بینی نمودند می‌توان نتیجه گیری کرد که الگوی فوق در تبیین رفتار غربالگری سرطان دهانه رحم چندان کارآمد نبوده و پیشنهاد می‌گردد که کارآمدی سایر الگوهای آموزشی در پیش بینی این رفتار مورد بررسی قرار گیرد. بعلاوه سازه‌های موانع درک شده، منافع درک شده و آگاهی در برنامه ریزی‌های مداخله‌ای مورد توجه بیشتری قرار گیرند .

Title: بررسی رعایت احتیاط های همه جانبه و عوامل موثر بر آن در درمانگاههای اورژانس بیمارستانهای منتخب دانشگاه علوم پزشکی مازندران

http://tbj.ssu.ac.ir/article-1-464-fa.html

مقدمه: در سال 1994 مرکز کنترل و پیشگیری بیماریهای آمریکا برای به حداقل رساندن احتمال آلودگی کارکنان مراقبتهای بهداشتی و درمانی با عوامل بیماریزای منتقل شونده با خون احتیاطهای همه جانبه را تدوین کرده است، لذا این پژوهش برای مشخص نمودن کیفیت و نحوه اجرای آن در درمانگاههای اورژانس بیمارستانهای منتخب دانشگاه علوم پزشکی مازندران انجام گرفت تا راهکارها و پیشنهادهای مناسب را در جهت زمینه های ترویج به کارگیری این احتیاطهای عمومی ارائه نماید.   روش بررسی: این مطالعه از نوع مقطعی(توصیفی-تحلیلی) و ابزار جمع آوری اطلاعات چک لیست بوده است. حجم نمونه برابر با220 نفر می باشد. داده ها پس از ورود در نرم افزار SPSS با بهره گیری از آمار توصیفی (جدول فراوانی، میانگین، انحراف معیار) و آزمونهای استنباطی (آزمون کای دو و رگرسیون لجستیک) تجزیه و تحلیل شده است.   یافته ها: 5/45 % از افراد مورد مطالعه مرد و 5/54% زن بودند. میزان رعایت کلی احتیاطهای همه جانبه در مردان و زنان به ترتیب 1/48% و 6/58% بوده است. براساس نتایج آزمون کای دو، تفاوت های مشاهده شده دررعایت کلی احتیاط های همه جانبه از نظر جنس، سابقه خدمت، رسته خدمتی، گروه سنی، شرکت در جلسات آموزشی وموجودبودن لوازم وتجهیزات معنی دار بوده است ( 05/0> P ).    نتیجه گیری: یافته های مطالعه نشان داد میزان رعایت کلی احتیاط های همه جانبه توسط پرسنل درمانگاه اورژانس در سطح متوسط بوده است در حالی که خطر انتقال ویروس هپاتیت B و HIV در این گونه تماسها بسیار زیاد می باشد. لذاپیاده سازی استانداردهای کنترل عفونت، اقدامات لازم در جهت ارتقاء انطباق کارکنان با مسائل حفاظت فردی، کنترل های مهندسی، رویکردهای شناختی، استراتژیهای اصلاح رفتار و ترکیب کردن مبانی نظری با تجربه های آموزشی به منظور ارتقاء مهارتها برای بکارگیری اصول استاندارد احتیاط های همه جانبه ضرورت دارد.

Title: بررسی رابطه بین رضایت شغلی با رضایت زناشویی کارکنان شاغل در سازمان بهزیستی شهرستان یزد

http://tbj.ssu.ac.ir/article-1-465-fa.html

مقدمه : رضایت شغلی می‌تواند از عوامل مهم و تأثیر گذار بر رضایت زناشویی باشد لذا در بین تمامی نهادها، سازمان‌ها و موسسات اجتماعی خانواده نقش و اهمیتی ویژه داشته و همه زوج‌ها به دنبال آن هستند که از زندگی زناشویی لذت ببرند و احساس رضایت داشته باشند نهایتاً رضایت شغلی بخش مهمی از رضایت زندگی محسوب می‌شود. پژوهش حاضر به منظور شناسایی رابطه ی بین رضایت شغلی و رضایت زناشویی کارکنان شاغل در سازمان بهزیستی شهرستان یزد در سال 1390 صورت گرفته است.   روش بررسی: روش این پژوهش توصیفی،تحلیلی، مقطعی می باشد. جامعه آماری شامل کلیه پرسنل شاغل در سازمان بهزیستی شهرستان یزد در سال 1390 که جمعاً 112 نفربوده واز این تعداد 102 نفر متاهل می باشند که از طریق سرشماری انتخاب شدند. ابزار جمع آوری اطلاعات پرسشنامه رضایت شغلی مینه سوتا وپرسشنامه رضایت زناشویی اینریچ بود.که روایی و پایایی انها در مطالعات قبلی تایید گردیده است تجزیه و تحلیل داده ها با استفاده از آمار توصیفی و استنباطی و به کمک نرم افزار spss win 13 انجام شد. برای تجزیه و تحلیل داده ها متناسب با فرضیه و سؤالات تحقیق از همبستگی اسپیرمن و پیرسون استفاده گردید.   یافته‌ها: نتایج نشان داد که 7/63 در صد شاغلین را زن و 3/36در صد را مرد تشکیل می دهند و بیشترین مدرک تحصیلی آنها لیسانس می باشد. میزان رضایت شغلی و رضایت زناشویی در دو جنس تفاوت داشت .بین سن و رضایت شغلی ورضایت زناشویی، بین سنوات خدمت و سطح تحصلات با رضایت شغلی رابطه وجود دارد.اما هیچکدام از انها معنی دار نمیباشد و در مابقی متغیرها رابطه ای وجود نداشت.   نتیجه‌گیری : نتایج نشان داد که بعضی از ویژگی‌های شخصیتی با رضایت شغلی و رضایت زناشویی رابطه دارند که توجه و دقت بیشتری در خصوص ویژگی‌های شخصیتی افراد برای استخدام مناسب را می‌طلبد تا بتوان تؤاماً رضایت شغلی و رضایت زناشویی را ارتقاء بخشید.

Title: بررسی علل انتخاب بیمارستان دولتی یا خصوصی برای دریافت مراقبت سلامت توسط بیماران در بیمارستان های شهر تبریز در سال 1388

http://tbj.ssu.ac.ir/article-1-466-fa.html

مقدمه: با افزایش چشمگیر موسسات و بیمارستان های خصوصی، بازار مراقبت سلامت به یک بازار رقابتی تبدیل شده و عوامل مختلفی بر انتخاب بیمارستان توسط بیمار برای دریافت خدمات موثر شده است. این پژوهش با هدف شناسایی عوامل موثر بر انتخاب بیمارستان (دولتی و خصوصی) از دیدگاه بیماران طراحی و اجرا گردید.   روش بررسی: این پژوهش یک مطالعه توصیفی - تحلیلی بود که جامعه آماری آن را بیماران بستری بیمارستان دولتی امام رضا(ع) و بیمارستان خصوصی شمس تبریز تشکیل می داد. تعداد 376 نفر نمونه با استفاده از روش تخصیص متناسب بین دو بیمارستان تقسیم گردید. ابزار گردآوری داده ها، پرسشنامه ای خود ساخته ای بود و داده ها با استفاده از آزمون های آماری مجذور کای( Chi-Square ) ، تی مستقل ( T –Test ) تحلیل گردید.   یافته ها: یافته ها نشان می دهند که عواملی از قبیل مراجعه از طریق اورژانس،توصیه پزشک معالج ، درآمد خانوار ،نوع بیمه ،کیفیت بیمارستان ، واشتغال افراد خانواردر بیمارستان ، هزینه خدمات ارائه شده در بیمارستان و آموزش ارائه شده به بیمار درمورد بیماری از عوامل تاثیر گذار بر انتخاب بیمارستان می باشند.   نتیجه گیری: هر چند بر اساس نتایج مطالعه عوامل مختلفی در انتخاب بیمارستان دولتی یا خصوصی موثر بودند، ولی بالا بودن درصد مراجعین اورژانسی به بیمارستان دولتی امام رضا(ع) و نقش بالای توصیه پزشکان معالج در انتخاب بیمارستان به علت انحصاری بودن پزشکان در مراکز آموزشی درمانی باعث گردید که عملا" بیماران نقشی در انتخاب نداشته و نقش عوامل مختلف در انتخاب بیمارستان کم رنگ تر شود

Title: بررسی کارایی پامیس اصلاح شده در حذف نیترات از محلول های آبی: مطالعه ایزوترم و سینتیک جذب

http://tbj.ssu.ac.ir/article-1-467-fa.html

مقدمه : آلودگی آب‌های زیرزمینی و سطحی به نیترات در بسیاری از مناطق دنیا به صورت یک مشکل جدی مطرح است. بالا بودن غلظت نیترات در آب برای کودکان مخاطره آمیز بوده و باعث ایجاد بیماری متهموگلوبینمیا می‌گردد . هدف از این پژوهش بررسی کارائی حذف نیترات با استفاده از کانی پامیس اصلاح شده با آهن و تعیین سینتیک و ایزو ترم های جذب می‌باشد.   روش بررسی: این تحقیق یک مطالعه تجربی بوده است که در مقیاس آزمایشگاهی به صورت ناپیوسته و در شرایط آزمایشگاهی ) دمای ‍ C ° 25 ( انجام گرفت. پارامتر های مختلفی از جمله غلظت اولیه محلول، زمان تماس و pH بررسی شد. در این پژوهش از دو مدل ایزو ترم فروندلیخ و لانگمایر جهت بررسی واکنش های جذب و محاسبه ثابت تعادل استفاده گردید. غلظت باقیمانده نیترات به وسیله دستگاه اسپکتوفتومتر UV/VIS ( Shimadzo-1700, Japan ) در طول موج‌های 220و 275 نانومتر اندازه گیری شد.   یافته‌ها: نتایج حاصل از مطالعات ایزوترمی نشان داد که حذف نیترات از معادله فروندلیخ ( 998/0 R2> ) پیروی می کند. در این مطالعه ضریب چسبندگی14/0 Kf = و مقدار نیترات جذب شده به ازای واحد جرم پامیس اصلاح شده برابر mg/g 65/0= qm(mg/g) می باشد. معادله شبه درجه دوم بهترین سازگاری را با توجه به داده های سنتیکی دارد.   نتیجه گیری : به طور کلی نتایج حاصل از این پژوهش نشان داد که می‌توان از پامیس اصلاح شده با آهن به عنوان جاذب موثر در جهت حذف نیترات از محلول‌های آبی استفاده کرد.

Title: تعیین میزان آنتی بادی هپاتیت ب(HBS ) در بین پرسنل بهداشتی بیمارستان سوانح سوختگی شهید صدوقی یزد در سال 1390

http://tbj.ssu.ac.ir/article-1-468-fa.html

مقدمه : هپاتیت ب یکی از مهمترین مشکلات بهداشتی است در دنیا بیش از 400میلیون نفر با این ویروس به حالت ناقل یا بیمار آلوده اند.پرسنل بهداشتی در خطر ابتلای بیشتری برای بیماریهای منتقله از راه خون شامل هپاتیت ب می باشند .هدف از این مطالعه تعیین میزان آنتی بادی HBS بین پرسنل بهداشتی بیمارستان سوانح سوختگی شهید صدوقی یزد می باشد.   روش بررسی:این مطالعه توصیفی مقطعی شامل 52 نفر از پرسنل واکسینه شده بیمارستان مذکور بود . آنتی بادی HBS توسط روش الیزا بر روی نمونه های خون سنجیده شد. تیتر بیش از ml / Iu 10 بعنوان نتیجه مثبت در نظر گرفته شد. داده ها با استفاده از نرم افزار(16) SPSS و آنالیز داده های توصیفی و تست دقیق فیشر مورد ارزیابی واقع شد.   یافته ها : از بین 52 پرسنل واکسینه شده ، 4/90% افراد تیتر مثبت و 6/9% تیتر منفی داشتند. زنان تیتر بالاتری نسبت به مردان داشتند،( 96% در برابر 2/85%) ارتباط آماری بین سن ، جنس ، نظم بین نوبت های واکسیناسیون و تیتر آنتی بادی HBS معنی دار نبود. ( 5 0/0 > P ) اما ارتباط بین مدت زمان گذشتن از آخرین نوبت واکسن و تیتر آنتی بادی معنی دار بود ( 46 0/0 = P ).   نتیجه گیری: 90% پرسنل بیمارستان علیه ویروس هپاتیت ب ایمنی داشتند نظر به اهمیت پرسنل بهداشتی در انتقال عفونتهای منتقله از راه خون ، تزریق دوز یاد آور واکسن هپاتیت ب باید به افرادی که تیتر آنتی بادی آنها قابل قبول نیست انجام شود.

Title: مروری بر زنبور درمانی در ایران وجهان

http://tbj.ssu.ac.ir/article-1-470-fa.html

مقدمه: انسان در طول تاریخ همواره در معرض عوامل تهدید کننده متعدد پاتوژ‍ژن و غیر پاتوژن قرار داشته و از راهها و عوامل مختلفی جهت درمان و کنترل آنها استفاده نموده است، یکی از این روش ها استفاده از زنبور و محصولات آن بوده است. سابقه استفاده از زنبور عسل و محصولات آن در درمان انواع بیماریها به شناخت انسان از مزایای این حشره بر می گردد.   روش بررسی: این مقاله یک مطالعه مروری است. برای انجام این تحقیق مطالب علمی مرتبط با موضوع تحقیق از منابع و سایت های معتبر علمی جمع آوری و مورد مطالعه قرار گرفت.   یافته ها: محصولات متنوعی نظیر عسل، موم، نیش، گرده، ژل رویال (سلطنتی)، پروپولیس و آپیلارنیل از جمله موادی هستند که در درمان بیماریهای مختلفی مانند آلرژی ها، بیماری های خونی، گوارشی، قلبی، ریوی، مفاصل، کلیوی، عصبی ( MS )، متابولیکی (دیابت )، داخلی، گوش، حلق، بینی و چشم، مقاربتی، نقص ایمنی (ایدز)، خونی، سرطان،روانی،پوستی ،استخوانی و بیماریهای حاصل از عوامل بیماریزا مثل ویروسها و ژیاردیاها، تریکوموناس ها، شاگاس و مالاریا استفاده می شوند.   نتیجه گیری: این مقاله مروری است بر برخی از جدیدترین دستاوردهای حاصل از زنبور عسل و محصولات آن که در کنترل و درمان بیماری ها در ایران و جهان به کار می رود.

Title: بررسی تاثیر آموزش از طریق بحث گروهی بر ارتقاء آگاهی بهداشت بلوغ جسمی دختران نوجوان ساکن مراکز شبانه روزی بهزیستی شهر تهران

http://tbj.ssu.ac.ir/article-1-469-fa.html

مقدمه: سازمان جهانی بهداشت نیازهای آموزشی زنان و دختران را به عنوان یک اولویت مشخص کرده است، بنابراین ضرورت انجام مطالعه پیرامون بهداشت نوجوان و نقطه عطف آن یعنی بهداشت بلوغ دختران، امری بدیهی است.این پژوهش با هدف تعیین تاثیر آموزش از طریق بحث گروهی بر ارتقاء آگاهی بهداشت بلوغ جسمی دختران نوجوان ساکن مراکز شبانه روزی بهزیستی شهر تهران انجام گرفت.   روش بررسی: پژوهش حاضر یک مطالعه مداخله ای نیمه تجربی (یک گروهی) می باشد، این مطالعه روی دختران 19-12 سال ساکن مراکز شبانه روزی بهزیستی شهر تهران انجام شد، همه افراد واجد شرایط(61 نفر) وارد مطالعه شدند. ابزارگردآوری اطلاعات پرسشنامه شامل دو قسمت خصوصیات دموگرافیک و سوالات آگاهی در خصوص بهداشت بلوغ جسمی بود که قبل و یک ماه پس از مداخله تکمیل گردید. مداخله آموزشی به مدت 3 ماه انجام گرفت، اطلاعات حاصل جمع آوری و توسط نرم افزار 16 SPSS با استفاده از آزمون آماری ویلکاکسون مقایسه شدند.   یافته ها: پس از مداخله آموزشی در مقایسه با قبل از آن میانگین نمره آگاهی از 7/4 ± 85/12 به 17/2 ± 01/22 افزایش یافت که با توجه به نتایج آزمون آماری ویلکاکسون این تغییر نمره از لحاظ آماری معنی دار بود(001/0 P < ).   نتیجه گیری: مداخله آموزشی از طریق بحث گروهی تاثیر مثبتی در بهبود آگاهی افراد مورد مطالعه در خصوص بهداشت بلوغ جسمی داشت، لذا شایسته است از این روش در آموزش مسائل مربوط به دوران بلوغ دختران نوجوان بهره گرفته شود.
